# Bis(acetyl­acetonato-κ^2^ *O*,*O*′)bis­(pyridine-κ*N*)nickel(II) dihydrate

**DOI:** 10.1107/S1600536813002699

**Published:** 2013-01-31

**Authors:** Mehdi Boutebdja, Adel Beghidja, Chahrazed Beghidja

**Affiliations:** a Unité de Recherche de Chimie de l’Environnement et, Moléculaire Structurale (CHEMS), Faculté des Sciences Exactes, Département de Chimie, Université de Constantine 1, 25000 Constantine, Algeria

## Abstract

The title compound, [Ni(C_5_H_7_O_2_)_2_(C_5_H_5_N)_2_]·2H_2_O, crystallizes with two half-mol­ecules in the asymmetric unit. The Ni^II^ ion of each unique complex mol­ecule lies on an inversion centre and has an octa­hedral coordination geometry. The crystal structure features weak O—H⋯O hydrogen bonds, which form chains running parallel to the *a* axis.

## Related literature

For the structures of octa­hedral complexes of the type [*M*(acac)_2_(*L*)_2_]_2_ (*M* = Ni; acac = acetyl­acetonate, 1,3-diphenyl-1,3 propane­dianato; *L* = pyridine, 3-cyano­pyridine, 4-cyano­pyridine, 3-methyl­pyridine, 2-methyl­pyridine, 4-pyridyl­tetra­thia­fulvalene), see: Elder (1968 ▶); Zukerman-Schpector *et al.* (2000 ▶, 2007 ▶); Wang *et al.* (2006 ▶); Soldatov *et al.* (2001 ▶).
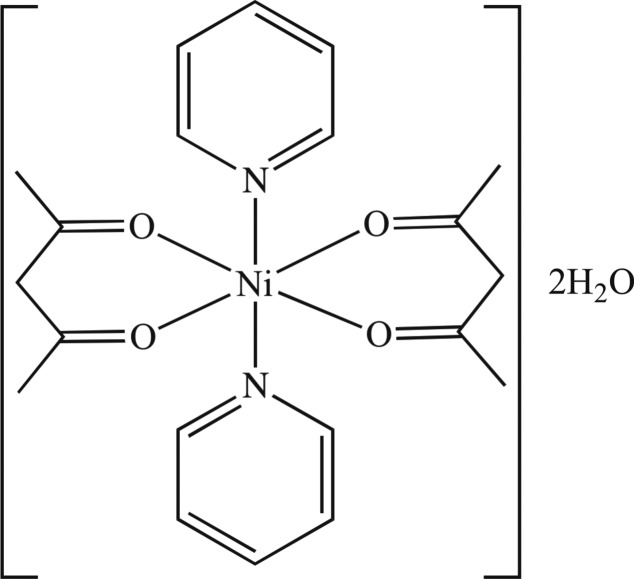


## Experimental

### 

#### Crystal data

[Ni(C_5_H_7_O_2_)_2_(C_5_H_5_N)_2_]·2H_2_O
*M* *_r_* = 451.13
Monoclinic, 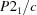

*a* = 16.362 (5) Å
*b* = 14.476 (5) Å
*c* = 9.543 (5) Å
β = 91.510 (5)°
*V* = 2259.5 (16) Å^3^
*Z* = 4
Mo *K*α radiation
μ = 0.89 mm^−1^
*T* = 296 K
0.15 × 0.12 × 0.10 mm

#### Data collection

Bruker APEXII CCD diffractometer
55078 measured reflections
5587 independent reflections
4298 reflections with *I* > 2σ(*I*)
*R*_int_ = 0.018

#### Refinement

*R*[*F* ^2^ > 2σ(*F* ^2^)] = 0.034
*wR*(*F* ^2^) = 0.103
*S* = 1.05
5587 reflections
269 parameters
6 restraints
H-atom parameters constrained
Δρ_max_ = 0.22 e Å^−3^
Δρ_min_ = −0.25 e Å^−3^

### 

Data collection: *APEX2* (Bruker, 2006 ▶); cell refinement: *SAINT* (Bruker, 2006 ▶); data reduction: *SAINT*; program(s) used to solve structure: *SHELXS97* (Sheldrick, 2008 ▶); program(s) used to refine structure: *SHELXL97* (Sheldrick, 2008 ▶); molecular graphics: *ATOMS* (Dowty, 1995 ▶); software used to prepare material for publication: *WinGX* (Farrugia, 2012 ▶).

## Supplementary Material

Crystal structure: contains datablock(s) global, I. DOI: 10.1107/S1600536813002699/kj2219sup1.cif

Structure factors: contains datablock(s) I. DOI: 10.1107/S1600536813002699/kj2219Isup2.hkl

Additional supplementary materials:  crystallographic information; 3D view; checkCIF report

## Figures and Tables

**Table 1:** Selected bond lengths (Å)

Ni1—O1	2.0427 (17)
Ni1—O2	2.0407 (16)
Ni1—N1	2.1039 (19)
Ni2—N2	2.126 (2)
Ni2—O3	2.0299 (16)
Ni2—O4	2.0297 (17)

**Table 2:** Hydrogen-bond geometry (Å, °)

*D*—H⋯*A*	*D*—H	H⋯*A*	*D*⋯*A*	*D*—H⋯*A*
O2*W*—H1*W*⋯O3	0.84	2.10	2.926 (3)	166
O2*W*—H2*W*⋯O1*W*	0.86	2.46	3.092 (4)	131
O1*W*—H11*W*⋯O2	0.83	2.45	2.908 (3)	116
O1*W*—H22*W*⋯O1^i^	0.84	2.07	2.896 (3)	169

Symmetry code: (i) 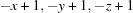.

**Fig. 1.**
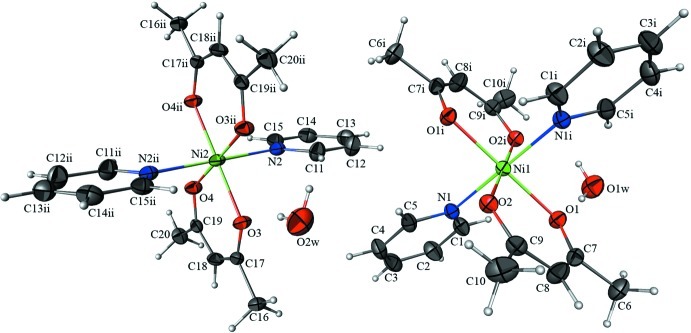
View of the title compound with the atom numbering scheme. Displacement ellipsoids for non-H atoms are drawn at the 50% probability level. symmetry code: i = 1 - x,1 - y,1 - z; ii = -x,1 - y,1 - z.

**Fig. 2.**
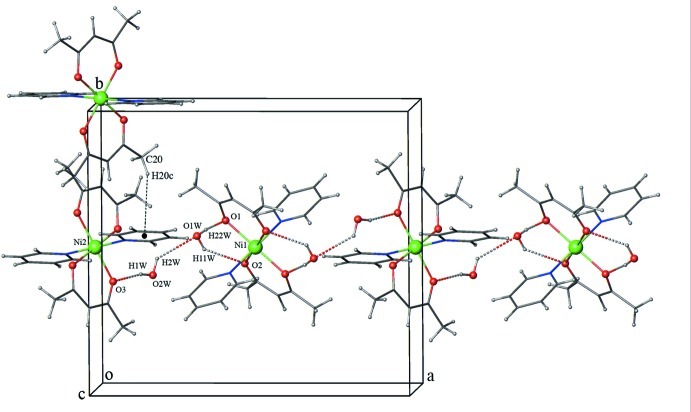
View down c of the crystal packing of the title compound showing the hydrogen bonds and C—H···π interactions as dashed lines.

## supplementary crystallographic information

### Comment

The crystal structure of the title compound Ni(acac)_2_(py)_2_.2H_2_O, where acac = acetylacetonate and py = pyridine, is composed of two crystallographically independent Ni complexes with similar geometry and with the Ni(II) atoms located on inversion centres as well as two water molecules located on general positions (Fig. 1). Both Ni(II) atoms have octahedral coordination geometry with the basal plane defined by the four oxygen atoms of the chelating acac (O1, O1^i^, O2, O2^i^) and (O3, O3^ii^, O4, O4^ii^) for Ni1 and Ni2 respectively, the axial positions are occupied by pyridine N atoms. Related structures have been reported previously in literature: the structure published by Elder (1968) is the anhydrate of the title compound, Zukerman-Schpector *et al.* (2007) published a methyl pyridine derivative, and the 3 and 4-cyanopyridine derivatives were also reported by Zukerman-Schpector and co-workers (2000). Other structures have been obtained with derivatives of acac: 1,3-diphenyl-1,3-propanedionato (Soldatov, *et al.*, (2001)) and 4-pyridyltetrathiafulvalene (Wang, *et al.*, (2006)). All the bond lengths and angles are in the normal range (Elder *et al.*, 1968; Zukerman-Schpector *et al.*, 2000; Wang *et al.*, 2006). The two independent units are linked to each other by weak O—H···O hydrogen bonds (see Table 2 for geometric details) which form a one-dimensional chain, running parallel to a as shown in Fig. 2. Another feature in this crystal structure is the presence of C—H···π interactions, which further stabilize the crystal packing. These C—H···π interactions are present between the C—H group of acac and an adjacent pyridine rings with a H···π distance of 3.183 Å (Fig. 2).

### Experimental

An amount of 0.075 g (0.5 mmol) of mercapto succinic acid was dissolved in 2 ml of pyridine and 0,1374 g (0.5 mmol) of nickel(II) acetylacetonate was dissolved in 10 ml methanol. The two solutions were mixed and stirred for 15 min. The resulting solution was allowed to stand at room temperature. After several days X-ray quality blue crystals were obtained. A fragment cut from a larger crystal was used for data collection.

### Refinement

Water hydrogen atoms were tentatively found in the difference density Fourier map and were refined with an isotropic displacement parameter 1.5 that of the adjacent oxygen atom. The O—H distances were restrained to be 0.9 Å within a standard deviation of 0.01 with *U*_iso_(H) = 1.5 *U*_eq_(O) and the H···H contacts were restraint to 1.40 Å with a standard deviation of 0.02. All other Hydrogen atoms were placed in calculated positions with C—H distances of 0.93–0.96 Å for aromatic H atoms with *U*_iso_(H) = 1.2 *U*_eq_(C).

### Crystal data

**Table:**

[Ni(C_5_H_7_O_2_)_2_(C_5_H_5_N)_2_]·2H_2_O	*F*(000) = 952
*M**_r_* = 451.13	*D*_x_ = 1.326 Mg m^−^^3^
Monoclinic, *P*2_1_/*c*	Mo *K*α radiation, λ = 0.71073 Å
Hall symbol: -P 2ybc	Cell parameters from 9929 reflections
*a* = 16.362 (5) Å	θ = 2.6–28.3°
*b* = 14.476 (5) Å	µ = 0.89 mm^−^^1^
*c* = 9.543 (5) Å	*T* = 296 K
β = 91.510 (5)°	Block, blue
*V* = 2259.5 (16) Å^3^	0.15 × 0.12 × 0.10 mm
*Z* = 4	

### Data collection

**Table:**

Bruker APEXII CCD diffractometer	4298 reflections with *I* > 2σ(*I*)
Radiation source: sealed tube	*R*_int_ = 0.018
Graphite monochromator	θ_max_ = 28.3°, θ_min_ = 1.2°
Detector resolution: 18.4 pixels mm^-1^	*h* = −21→21
φ and ω scans	*k* = −18→19
55078 measured reflections	*l* = −12→12
5587 independent reflections	

### Refinement

**Table:**

Refinement on *F*^2^	Primary atom site location: structure-invariant direct methods
Least-squares matrix: full	Secondary atom site location: difference Fourier map
*R*[*F*^2^ > 2σ(*F*^2^)] = 0.034	Hydrogen site location: inferred from neighbouring sites
*wR*(*F*^2^) = 0.103	H-atom parameters constrained
*S* = 1.05	*w* = 1/[σ^2^(*F*_o_^2^) + (0.0451*P*)^2^ + 0.9372*P*] where *P* = (*F*_o_^2^ + 2*F*_c_^2^)/3
5587 reflections	(Δ/σ)_max_ < 0.001
269 parameters	Δρ_max_ = 0.22 e Å^−^^3^
6 restraints	Δρ_min_ = −0.25 e Å^−^^3^

### Special details

**Table:**

Geometry. Bond distances, angles *etc*. have been calculated using the rounded fractional coordinates. All su's are estimated from the variances of the (full) variance-covariance matrix. The cell e.s.d.'s are taken into account in the estimation of distances, angles and torsion angles
Refinement. Refinement on *F*^2^ for ALL reflections except those flagged by the user for potential systematic errors. Weighted *R*-factors *wR* and all goodnesses of fit *S* are based on *F*^2^, conventional *R*-factors *R* are based on *F*, with *F* set to zero for negative *F*^2^. The observed criterion of *F*^2^ > σ(*F*^2^) is used only for calculating -*R*-factor-obs *etc*. and is not relevant to the choice of reflections for refinement. *R*-factors based on *F*^2^ are statistically about twice as large as those based on *F*, and *R*-factors based on ALL data will be even larger.

### Fractional atomic coordinates and isotropic or equivalent isotropic displacement parameters (Å^2^)

**Table:**

	*x*	*y*	*z*	*U*_iso_*/*U*_eq_	
Ni1	0.50000	0.50000	0.50000	0.0388 (1)	
O1	0.59123 (8)	0.40479 (9)	0.52664 (13)	0.0504 (4)	
O2	0.47500 (7)	0.45218 (9)	0.30232 (12)	0.0478 (4)	
N1	0.41637 (9)	0.40607 (11)	0.58429 (15)	0.0458 (4)	
C1	0.43286 (13)	0.36179 (16)	0.7042 (2)	0.0623 (7)	
C2	0.38029 (16)	0.29902 (19)	0.7614 (3)	0.0771 (9)	
C3	0.30771 (15)	0.27972 (18)	0.6921 (3)	0.0771 (9)	
C4	0.29001 (13)	0.32532 (18)	0.5691 (2)	0.0703 (8)	
C5	0.34512 (11)	0.38751 (15)	0.5184 (2)	0.0549 (6)	
C6	0.68575 (14)	0.28756 (18)	0.4746 (3)	0.0733 (8)	
C7	0.61380 (11)	0.34840 (14)	0.43447 (19)	0.0502 (6)	
C8	0.57898 (14)	0.33964 (17)	0.3012 (2)	0.0663 (8)	
C9	0.51543 (12)	0.39087 (14)	0.24124 (18)	0.0509 (6)	
C10	0.49162 (19)	0.3735 (2)	0.0893 (2)	0.0821 (9)	
Ni2	0.00000	0.50000	0.50000	0.0454 (1)	
O3	0.05752 (8)	0.38202 (8)	0.44348 (15)	0.0548 (4)	
O4	−0.07630 (8)	0.43023 (9)	0.62668 (15)	0.0553 (4)	
N2	0.08437 (10)	0.51694 (10)	0.67131 (18)	0.0518 (5)	
C11	0.16473 (15)	0.51415 (17)	0.6544 (3)	0.0696 (8)	
C12	0.22079 (17)	0.5225 (2)	0.7630 (3)	0.0834 (10)	
C13	0.19364 (18)	0.53429 (18)	0.8970 (3)	0.0774 (9)	
C14	0.11092 (17)	0.53708 (17)	0.9161 (3)	0.0722 (9)	
C15	0.05890 (14)	0.52919 (14)	0.8021 (2)	0.0602 (7)	
C16	0.08741 (14)	0.22314 (14)	0.4275 (3)	0.0663 (8)	
C17	0.03892 (11)	0.30288 (11)	0.48564 (19)	0.0464 (5)	
C18	−0.02264 (13)	0.28323 (12)	0.5796 (2)	0.0552 (6)	
C19	−0.07502 (11)	0.34409 (12)	0.64369 (18)	0.0449 (5)	
C20	−0.13618 (13)	0.30541 (16)	0.7449 (2)	0.0629 (7)	
O1W	0.33029 (12)	0.55261 (16)	0.2056 (2)	0.0998 (8)	
O2W	0.18999 (13)	0.41332 (17)	0.2490 (2)	0.1108 (9)	
H1	0.48220	0.37390	0.75120	0.0750*	
H2	0.39370	0.27000	0.84590	0.0930*	
H3	0.27140	0.23680	0.72780	0.0930*	
H4	0.24100	0.31420	0.52040	0.0840*	
H5	0.33240	0.41800	0.43490	0.0660*	
H6A	0.68450	0.27370	0.57290	0.1100*	
H6B	0.73570	0.31920	0.45470	0.1100*	
H6C	0.68270	0.23120	0.42180	0.1100*	
H8	0.60090	0.29390	0.24500	0.0800*	
H10A	0.43520	0.38940	0.07350	0.1230*	
H10B	0.49960	0.30950	0.06770	0.1230*	
H10C	0.52500	0.41070	0.03030	0.1230*	
H11	0.18390	0.50610	0.56430	0.0840*	
H12	0.27650	0.52020	0.74620	0.1000*	
H13	0.23040	0.54020	0.97250	0.0930*	
H14	0.09040	0.54420	1.00550	0.0870*	
H15	0.00300	0.53250	0.81620	0.0720*	
H16A	0.12230	0.24540	0.35560	0.0990*	
H16B	0.05050	0.17770	0.38880	0.0990*	
H16C	0.12020	0.19580	0.50150	0.0990*	
H18	−0.02930	0.22120	0.60200	0.0660*	
H20A	−0.18290	0.34540	0.74740	0.0940*	
H20B	−0.11120	0.30130	0.83690	0.0940*	
H20C	−0.15310	0.24500	0.71440	0.0940*	
H11W	0.35400	0.50780	0.17200	0.1500*	
H22W	0.35500	0.57180	0.27800	0.1500*	
H1W	0.15010	0.41390	0.30310	0.1660*	
H2W	0.20310	0.47070	0.25810	0.1660*	

### Atomic displacement parameters (Å^2^)

**Table:**

	*U*^11^	*U*^22^	*U*^33^	*U*^12^	*U*^13^	*U*^23^
Ni1	0.0400 (2)	0.0425 (2)	0.0336 (2)	0.0005 (1)	−0.0026 (1)	−0.0044 (1)
O1	0.0492 (7)	0.0560 (7)	0.0456 (6)	0.0076 (6)	−0.0067 (5)	−0.0061 (6)
O2	0.0511 (7)	0.0537 (7)	0.0383 (6)	0.0006 (6)	−0.0061 (5)	−0.0068 (5)
N1	0.0468 (8)	0.0504 (8)	0.0402 (7)	−0.0042 (6)	−0.0011 (6)	−0.0032 (6)
C1	0.0618 (12)	0.0696 (13)	0.0550 (11)	−0.0134 (10)	−0.0092 (9)	0.0098 (10)
C2	0.0854 (17)	0.0819 (17)	0.0640 (13)	−0.0222 (13)	0.0003 (12)	0.0185 (12)
C3	0.0743 (15)	0.0784 (16)	0.0795 (15)	−0.0278 (13)	0.0189 (12)	0.0000 (13)
C4	0.0523 (11)	0.0885 (16)	0.0700 (13)	−0.0208 (11)	0.0018 (10)	−0.0099 (12)
C5	0.0479 (10)	0.0685 (12)	0.0480 (9)	−0.0059 (9)	−0.0016 (8)	−0.0052 (9)
C6	0.0612 (13)	0.0821 (16)	0.0767 (14)	0.0246 (12)	0.0031 (11)	0.0002 (12)
C7	0.0452 (9)	0.0539 (10)	0.0517 (10)	0.0038 (8)	0.0076 (7)	−0.0003 (8)
C8	0.0707 (13)	0.0781 (15)	0.0503 (11)	0.0186 (11)	0.0041 (9)	−0.0187 (10)
C9	0.0597 (11)	0.0565 (11)	0.0366 (8)	−0.0056 (9)	0.0031 (7)	−0.0058 (8)
C10	0.117 (2)	0.0896 (17)	0.0392 (10)	0.0126 (15)	−0.0071 (11)	−0.0138 (11)
Ni2	0.0479 (2)	0.0291 (2)	0.0602 (2)	0.0021 (1)	0.0166 (2)	0.0029 (1)
O3	0.0569 (8)	0.0353 (6)	0.0730 (9)	0.0058 (5)	0.0172 (6)	−0.0004 (6)
O4	0.0558 (7)	0.0408 (7)	0.0703 (8)	−0.0018 (6)	0.0198 (6)	0.0068 (6)
N2	0.0554 (9)	0.0389 (7)	0.0615 (9)	0.0026 (6)	0.0120 (7)	0.0016 (7)
C11	0.0579 (13)	0.0834 (16)	0.0680 (14)	0.0098 (11)	0.0090 (10)	−0.0047 (11)
C12	0.0616 (14)	0.102 (2)	0.0864 (18)	0.0118 (14)	−0.0030 (13)	−0.0064 (15)
C13	0.0899 (18)	0.0682 (15)	0.0734 (15)	0.0006 (13)	−0.0124 (13)	−0.0001 (12)
C14	0.0941 (18)	0.0618 (13)	0.0613 (13)	−0.0074 (13)	0.0111 (12)	−0.0023 (11)
C15	0.0655 (13)	0.0493 (10)	0.0667 (13)	−0.0038 (9)	0.0165 (10)	−0.0018 (9)
C16	0.0661 (13)	0.0401 (10)	0.0920 (16)	0.0103 (9)	−0.0103 (11)	−0.0159 (10)
C17	0.0490 (9)	0.0346 (8)	0.0549 (10)	0.0015 (7)	−0.0133 (7)	−0.0053 (7)
C18	0.0684 (12)	0.0327 (8)	0.0644 (11)	−0.0072 (8)	−0.0026 (9)	0.0044 (8)
C19	0.0475 (9)	0.0425 (9)	0.0442 (9)	−0.0102 (7)	−0.0103 (7)	0.0058 (7)
C20	0.0660 (12)	0.0641 (13)	0.0586 (11)	−0.0177 (10)	0.0023 (9)	0.0164 (10)
O1W	0.0906 (13)	0.1176 (17)	0.0896 (13)	0.0192 (12)	−0.0296 (10)	−0.0028 (12)
O2W	0.1004 (15)	0.1241 (18)	0.1093 (16)	0.0189 (13)	0.0298 (12)	−0.0021 (13)

### Geometric parameters (Å, º)

**Table:**

Ni1—O1	2.0427 (17)	C1—H1	0.9300
Ni1—O2	2.0407 (16)	C2—H2	0.9300
Ni1—N1	2.1039 (19)	C3—H3	0.9300
Ni1—O1^i^	2.0427 (17)	C4—H4	0.9300
Ni1—O2^i^	2.0407 (16)	C5—H5	0.9300
Ni1—N1^i^	2.1039 (19)	C6—H6A	0.9600
Ni2—N2^ii^	2.126 (2)	C6—H6C	0.9600
Ni2—N2	2.126 (2)	C6—H6B	0.9600
Ni2—O3	2.0299 (16)	C8—H8	0.9300
Ni2—O4	2.0297 (17)	C10—H10B	0.9600
Ni2—O3^ii^	2.0298 (16)	C10—H10A	0.9600
Ni2—O4^ii^	2.0297 (17)	C10—H10C	0.9600
O1—C7	1.262 (2)	C11—C12	1.371 (4)
O2—C9	1.259 (2)	C12—C13	1.375 (4)
O3—C17	1.254 (2)	C13—C14	1.371 (4)
O4—C19	1.258 (2)	C14—C15	1.368 (4)
O1W—H22W	0.8400	C16—C17	1.514 (3)
O1W—H11W	0.8300	C17—C18	1.395 (3)
O2W—H2W	0.8600	C18—C19	1.383 (3)
O2W—H1W	0.8400	C19—C20	1.516 (3)
N1—C5	1.337 (2)	C11—H11	0.9300
N1—C1	1.333 (3)	C12—H12	0.9300
N2—C11	1.330 (3)	C13—H13	0.9300
N2—C15	1.338 (3)	C14—H14	0.9300
C1—C2	1.374 (4)	C15—H15	0.9300
C2—C3	1.373 (4)	C16—H16A	0.9600
C3—C4	1.371 (4)	C16—H16B	0.9600
C4—C5	1.371 (3)	C16—H16C	0.9600
C6—C7	1.511 (3)	C18—H18	0.9300
C7—C8	1.386 (3)	C20—H20B	0.9600
C8—C9	1.388 (3)	C20—H20C	0.9600
C9—C10	1.513 (3)	C20—H20A	0.9600
			
Ni1···O1W	3.972 (3)	N2···O4^ii^	2.944 (3)
Ni1···O1W^i^	3.972 (3)	N2···O3	2.947 (3)
Ni1···H22W	3.3100	N2···O3^ii^	2.932 (3)
Ni1···H22W^i^	3.3100	C2···C10^vi^	3.523 (4)
Ni2···H1W^ii^	3.3700	C2···C9^vi^	3.536 (4)
Ni2···H1W	3.3700	C4···C11	3.525 (4)
O1···N1	2.928 (2)	C9···C2^v^	3.536 (4)
O1···O2	2.908 (2)	C10···C2^v^	3.523 (4)
O1···N1^i^	2.937 (3)	C11···C4	3.525 (4)
O1···O1W^i^	2.896 (3)	C1···H10B^vi^	3.0200
O1···O2^i^	2.867 (2)	C4···H2^v^	3.0800
O1···C9	2.970 (3)	C5···H2^v^	2.9300
O1···C5^i^	3.215 (3)	C7···H22W^i^	3.0100
O1···C1	3.195 (3)	C10···H11W	3.0900
O1W···Ni1	3.972 (3)	C14···H18^vii^	2.9800
O1W···O2	2.908 (3)	C15···H18^vii^	2.9700
O1W···O2W	3.092 (4)	C16···H4	2.9500
O1W···Ni1	3.972 (3)	C17···H1W	3.0200
O1W···O1^i^	2.896 (3)	C18···H20B^v^	2.9600
O2···O1W	2.908 (3)	C19···H16B^vi^	3.0900
O2···C7	2.976 (3)	H1···O2^i^	2.6700
O2···O1	2.908 (2)	H1···O1	2.8600
O2···C1^i^	3.088 (3)	H1W···O3	2.1000
O2···N1	2.957 (2)	H1W···C17	3.0200
O2···C5	3.143 (3)	H1W···Ni2	3.3700
O2···O1^i^	2.867 (2)	H1W···O4^ii^	2.6500
O2···N1^i^	2.905 (2)	H1W···H16A	2.5300
O2W···O1W	3.092 (4)	H1W···Ni2	3.3700
O2W···O4^ii^	3.180 (3)	H2···C4^vi^	3.0800
O2W···O3	2.926 (3)	H2···C5^vi^	2.9300
O3···N2^ii^	2.932 (3)	H2W···O4^ii^	2.7700
O3···O4	2.922 (2)	H2W···O1W	2.4600
O3···C19	2.979 (3)	H3···O2W^vi^	2.5600
O3···O2W	2.926 (3)	H4···C16	2.9500
O3···C11	3.256 (3)	H5···O2W	2.8900
O3···O4^ii^	2.818 (2)	H5···O1^i^	2.8700
O3···N2	2.947 (3)	H5···O2	2.7300
O3···C15^ii^	3.246 (3)	H6A···H8^vi^	2.3800
O4···N2	2.934 (3)	H6C···O1W^viii^	2.8600
O4···O3^ii^	2.818 (2)	H6C···H8	2.3100
O4···C17	2.983 (3)	H8···H10B	2.3500
O4···C15	3.091 (3)	H8···H6A^v^	2.3800
O4···O2W^ii^	3.180 (3)	H8···H6C	2.3100
O4···N2^ii^	2.944 (3)	H10A···H11W	2.3800
O4···O3	2.922 (2)	H10B···C1^v^	3.0200
O4···C11^ii^	3.120 (4)	H10B···H8	2.3500
O1···H22W^i^	2.0700	H11···O4^ii^	2.6600
O1···H5^i^	2.8700	H11W···O2	2.4500
O1···H1	2.8600	H11W···C10	3.0900
O1W···H6C^iii^	2.8600	H11W···H10A	2.3800
O1W···H20A^ii^	2.8700	H13···O1W^ix^	2.7300
O1W···H13^iv^	2.7300	H14···H15^x^	2.5700
O1W···H2W	2.4600	H15···H14^x^	2.5700
O2···H1^i^	2.6700	H15···O4	2.6500
O2···H22W	2.6200	H15···O3^ii^	2.9200
O2···H11W	2.4500	H16A···O2W	2.8700
O2···H5	2.7300	H16A···H1W	2.5300
O2W···H5	2.8900	H16B···C19^v^	3.0900
O2W···H3^v^	2.5600	H16B···H18	2.5300
O2W···H16A	2.8700	H18···C15^xi^	2.9700
O3···H1W	2.1000	H18···C14^xi^	2.9800
O3···H15^ii^	2.9200	H18···H16B	2.5300
O4···H15	2.6500	H18···H20C	2.3400
O4···H1W^ii^	2.6500	H20A···O1W^ii^	2.8700
O4···H11^ii^	2.6600	H20B···C18^vi^	2.9600
O4···H2W^ii^	2.7700	H20C···H18	2.3400
N1···O1	2.928 (2)	H22W···C7^i^	3.0100
N1···O2	2.957 (2)	H22W···Ni1	3.3100
N1···O1^i^	2.937 (3)	H22W···O2	2.6200
N1···O2^i^	2.905 (2)	H22W···Ni1	3.3100
N2···O4	2.934 (3)	H22W···O1^i^	2.0700
			
O1—Ni1—O2	90.81 (5)	C3—C2—H2	120.00
O1—Ni1—N1	89.82 (6)	C4—C3—H3	121.00
O1—Ni1—O1^i^	180.00	C2—C3—H3	121.00
O1—Ni1—O2^i^	89.19 (5)	C3—C4—H4	120.00
O1—Ni1—N1^i^	90.18 (6)	C5—C4—H4	120.00
O2—Ni1—N1	91.01 (5)	C4—C5—H5	119.00
O1^i^—Ni1—O2	89.19 (5)	N1—C5—H5	119.00
O2—Ni1—O2^i^	180.00	H6A—C6—H6B	109.00
O2—Ni1—N1^i^	88.99 (5)	H6A—C6—H6C	109.00
O1^i^—Ni1—N1	90.18 (6)	C7—C6—H6A	109.00
O2^i^—Ni1—N1	88.99 (5)	C7—C6—H6B	109.00
N1—Ni1—N1^i^	180.00	H6B—C6—H6C	110.00
O1^i^—Ni1—O2^i^	90.81 (5)	C7—C6—H6C	109.00
O1^i^—Ni1—N1^i^	89.82 (6)	C7—C8—H8	116.00
O2^i^—Ni1—N1^i^	91.01 (5)	C9—C8—H8	116.00
O4—Ni2—N2	89.80 (6)	H10A—C10—H10C	109.00
O3^ii^—Ni2—O4	87.92 (5)	H10B—C10—H10C	109.00
O4—Ni2—O4^ii^	180.00	C9—C10—H10C	109.00
O4—Ni2—N2^ii^	90.20 (6)	H10A—C10—H10B	110.00
O3^ii^—Ni2—N2	89.70 (6)	C9—C10—H10A	109.00
O4^ii^—Ni2—N2	90.20 (6)	C9—C10—H10B	109.00
N2—Ni2—N2^ii^	180.00	N2—C11—C12	123.3 (3)
O3^ii^—Ni2—O4^ii^	92.08 (5)	C11—C12—C13	119.2 (3)
O3^ii^—Ni2—N2^ii^	90.30 (6)	C12—C13—C14	118.1 (3)
O4^ii^—Ni2—N2^ii^	89.80 (6)	C13—C14—C15	119.2 (3)
O3—Ni2—O4	92.08 (5)	N2—C15—C14	123.4 (2)
O3—Ni2—N2	90.30 (6)	C16—C17—C18	118.30 (16)
O3—Ni2—O3^ii^	180.00	O3—C17—C18	125.29 (16)
O3—Ni2—O4^ii^	87.92 (5)	O3—C17—C16	116.41 (17)
O3—Ni2—N2^ii^	89.70 (6)	C17—C18—C19	128.36 (16)
Ni1—O1—C7	125.16 (12)	O4—C19—C20	116.07 (16)
Ni1—O2—C9	124.90 (11)	O4—C19—C18	125.66 (17)
Ni2—O3—C17	124.41 (12)	C18—C19—C20	118.27 (17)
Ni2—O4—C19	124.19 (12)	C12—C11—H11	118.00
H11W—O1W—H22W	111.00	N2—C11—H11	118.00
H1W—O2W—H2W	97.00	C13—C12—H12	120.00
Ni1—N1—C1	121.33 (13)	C11—C12—H12	120.00
Ni1—N1—C5	121.19 (13)	C14—C13—H13	121.00
C1—N1—C5	117.47 (17)	C12—C13—H13	121.00
Ni2—N2—C15	121.38 (14)	C13—C14—H14	120.00
C11—N2—C15	116.8 (2)	C15—C14—H14	120.00
Ni2—N2—C11	121.83 (16)	N2—C15—H15	118.00
N1—C1—C2	123.0 (2)	C14—C15—H15	118.00
C1—C2—C3	119.1 (2)	C17—C16—H16C	109.00
C2—C3—C4	118.4 (2)	H16A—C16—H16B	110.00
C3—C4—C5	119.5 (2)	C17—C16—H16B	109.00
N1—C5—C4	122.63 (18)	H16B—C16—H16C	110.00
C6—C7—C8	118.55 (19)	H16A—C16—H16C	109.00
O1—C7—C8	125.31 (18)	C17—C16—H16A	109.00
O1—C7—C6	116.13 (18)	C17—C18—H18	116.00
C7—C8—C9	127.9 (2)	C19—C18—H18	116.00
O2—C9—C10	115.84 (18)	C19—C20—H20B	109.00
O2—C9—C8	125.64 (17)	C19—C20—H20C	109.00
C8—C9—C10	118.52 (19)	H20A—C20—H20C	109.00
N1—C1—H1	118.00	H20B—C20—H20C	109.00
C2—C1—H1	119.00	H20A—C20—H20B	110.00
C1—C2—H2	120.00	C19—C20—H20A	109.00
			
O2—Ni1—O1—C7	2.88 (15)	Ni1—O1—C7—C6	176.78 (14)
N1—Ni1—O1—C7	93.89 (15)	Ni1—O1—C7—C8	−2.0 (3)
O2^i^—Ni1—O1—C7	−177.12 (15)	Ni1—O2—C9—C8	6.1 (3)
N1^i^—Ni1—O1—C7	−86.11 (15)	Ni1—O2—C9—C10	−173.57 (15)
O1—Ni1—O2—C9	−4.80 (15)	Ni2—O3—C17—C18	−1.4 (3)
N1—Ni1—O2—C9	−94.64 (15)	Ni2—O3—C17—C16	178.01 (14)
O1^i^—Ni1—O2—C9	175.20 (15)	Ni2—O4—C19—C20	178.97 (12)
N1^i^—Ni1—O2—C9	85.37 (15)	Ni2—O4—C19—C18	−0.4 (3)
O1—Ni1—N1—C1	49.00 (15)	C1—N1—C5—C4	−0.3 (3)
O2—Ni1—N1—C1	139.81 (15)	Ni1—N1—C1—C2	−179.04 (18)
O1^i^—Ni1—N1—C1	−131.00 (15)	Ni1—N1—C5—C4	178.68 (16)
O2^i^—Ni1—N1—C1	−40.19 (15)	C5—N1—C1—C2	0.0 (3)
O1—Ni1—N1—C5	−129.99 (15)	Ni2—N2—C11—C12	−178.3 (2)
O2—Ni1—N1—C5	−39.18 (15)	C15—N2—C11—C12	0.4 (3)
O1^i^—Ni1—N1—C5	50.01 (15)	C11—N2—C15—C14	−1.2 (3)
O2^i^—Ni1—N1—C5	140.82 (15)	Ni2—N2—C15—C14	177.59 (17)
O4^ii^—Ni2—N2—C15	145.01 (15)	N1—C1—C2—C3	0.7 (4)
O4—Ni2—O3—C17	1.15 (15)	C1—C2—C3—C4	−1.1 (4)
N2—Ni2—O3—C17	90.97 (15)	C2—C3—C4—C5	0.7 (4)
O4^ii^—Ni2—O3—C17	−178.85 (15)	C3—C4—C5—N1	0.0 (3)
N2^ii^—Ni2—O3—C17	−89.04 (15)	O1—C7—C8—C9	1.8 (4)
O3—Ni2—O4—C19	−0.32 (15)	C6—C7—C8—C9	−177.0 (2)
N2—Ni2—O4—C19	−90.61 (15)	C7—C8—C9—C10	175.5 (2)
O3^ii^—Ni2—O4—C19	179.68 (15)	C7—C8—C9—O2	−4.1 (4)
N2^ii^—Ni2—O4—C19	89.39 (15)	N2—C11—C12—C13	0.1 (4)
O3—Ni2—N2—C11	51.62 (16)	C11—C12—C13—C14	0.1 (4)
O4—Ni2—N2—C11	143.71 (16)	C12—C13—C14—C15	−0.8 (4)
O3^ii^—Ni2—N2—C11	−128.38 (16)	C13—C14—C15—N2	1.4 (4)
O4^ii^—Ni2—N2—C11	−36.29 (16)	O3—C17—C18—C19	0.5 (3)
O3—Ni2—N2—C15	−127.07 (15)	C16—C17—C18—C19	−178.9 (2)
O4—Ni2—N2—C15	−34.99 (15)	C17—C18—C19—C20	−178.79 (19)
O3^ii^—Ni2—N2—C15	52.93 (15)	C17—C18—C19—O4	0.5 (3)

Symmetry codes: (i) −*x*+1, −*y*+1, −*z*+1; (ii) −*x*, −*y*+1, −*z*+1; (iii) −*x*+1, *y*+1/2, −*z*+1/2; (iv) *x*, *y*, *z*−1; (v) *x*, −*y*+1/2, *z*−1/2; (vi) *x*, −*y*+1/2, *z*+1/2; (vii) −*x*, *y*+1/2, −*z*+3/2; (viii) −*x*+1, *y*−1/2, −*z*+1/2; (ix) *x*, *y*, *z*+1; (x) −*x*, −*y*+1, −*z*+2; (xi) −*x*, *y*−1/2, −*z*+3/2.

### Hydrogen-bond geometry (Å, º)

**Table:**

*D*—H···*A*	*D*—H	H···*A*	*D*···*A*	*D*—H···*A*
O2*W*—H1*W*···O3	0.84	2.10	2.926 (3)	166
O2*W*—H2*W*···O1*W*	0.86	2.46	3.092 (4)	131
O1*W*—H11*W*···O2	0.83	2.45	2.908 (3)	116
O1*W*—H22*W*···O1^i^	0.84	2.07	2.896 (3)	169
C3—H3···O2*W*^vi^	0.93	2.56	3.445 (4)	159

Symmetry codes: (i) −*x*+1, −*y*+1, −*z*+1; (vi) *x*, −*y*+1/2, *z*+1/2.
